# Supplementary material for: Therapeutic Potential of an Anti-CD44v6 Monoclonal Antibody in Xenograft Models of Colorectal and Gastric Cancer
Source: Cells. 2025 Nov 26;14(23):1873. doi: 10.3390/cells14231873 (PMC12691535; doi:10.3390/cells14231873)
Supplement: Supplementary file 1 [file cells-14-01873-s001.zip › cells-3959370-supplementary.pdf]

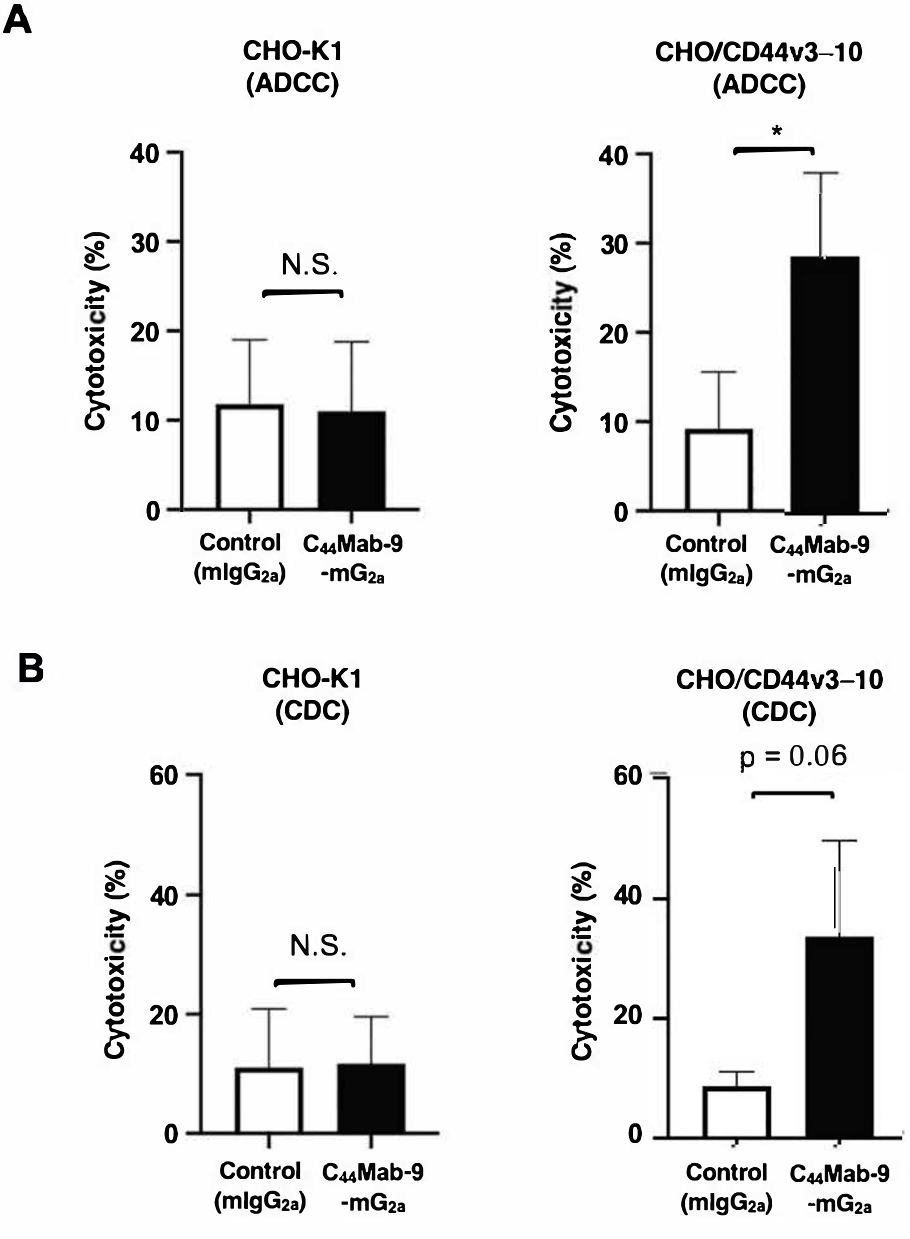

Supplementary Figure S1. ADCC and CDC against CHO-K1 and CHO/CD44v3–10 by C_{44}Mab-9-mG_{2a}. (**A**) Calcein AM-labeled target CHO-K1 and CHO/CD44v3–10 were incubated with the effector splenocytes in the presence of 100 μg/mL of C_{44}Mab-9-mG_{2a} or control mIgG_{2a}. (**B**) Calcein AM-labeled target CHO-K1 and CHO/CD44v3–10 were incubated with complements and 100 μg/mL of C_{44}Mab-9-mG_{2a} or control mIgG_{2a}. Following a 4-hour incubation, the Calcein release into the medium was measured. Values are shown as mean ± SEM. Asterisks indicate statistical significance (* $p < 0.05$; Two-tailed unpaired t test). N.S., not significant
